# Higher-order transient structures and the principle of dynamic connectivity in membrane signaling

**DOI:** 10.1073/pnas.2421280121

**Published:** 2024-12-31

**Authors:** Yuxi Zhang, Roderick MacKinnon

**Affiliations:** ^a^Laboratory of Molecular Neurobiology and Biophysics, The Rockefeller University, New York, NY 10065; ^b^HHMI, The Rockefeller University, New York, NY 10065

**Keywords:** self-assembly, higher-order transient structure, HOTS, membrane signaling, GPCR

## Abstract

We examine the role of higher-order transient structures (HOTS) in M2R regulation of GIRK channels. Electron microscopic membrane protein location maps show that both proteins form HOTS that exhibit a statistical bias to be near each other. Theoretical calculations and electrophysiological measurements suggest that channel activity is isolated near larger M2R HOTS. By invoking weak interactions that permit transient binding of M2R to M2R and GIRK to GIRK (*i-i* interactions) and M2R to GIRK (*i-j* interactions), the distribution patterns and electrophysiological properties of HL-1 cells are replicated in a reaction-diffusion simulation. We propose the principle of dynamic connectivity to explain communication between protein components of a membrane signaling pathway. Dynamic connectivity is mediated by weak, transient interactions between proteins. HOTS created by weak *i-i* interactions, and statistical biases created by weak *i-j* interactions promoted by the multivalence of HOTS, are the key elements of dynamic connectivity.

## Significance

Heart rate is controlled by neurotransmitter activation of various G-protein-coupled receptors in heart cell membranes. The messages to speed or slow are communicated with fidelity along separate “signal pathways” consisting of membrane proteins. In contrast to the hard-wired components of electronic devices, the membrane proteins in signal pathways contact each other transiently but sufficiently through specific protein–protein interactions. We call this kind of communication dynamic connectivity. A form of self-organization called higher-order transient structures lies at the heart of dynamic connectivity.

In the preceding paper, we examined the distribution of three G-protein-coupled receptors (GPCRs), one ion channel, and the enzyme adenylate cyclase (AC), all expressed naturally in the plasma membrane of a cardiac-derived HL-1 cell line (1). Our findings led us to hypothesize that these proteins each form clusters through self-oligomerization mediated by multiple weak interactions. We call these clusters higher-order transient structures (HOTS). This kind of self-assembly can give rise to a phase transition and the consequent production of larger, bulk phase clusters; however, HL-1 cells exhibit mainly HOTS. The question we address in this study is do HOTS play a functional role in signaling?

**Fig. 1.**
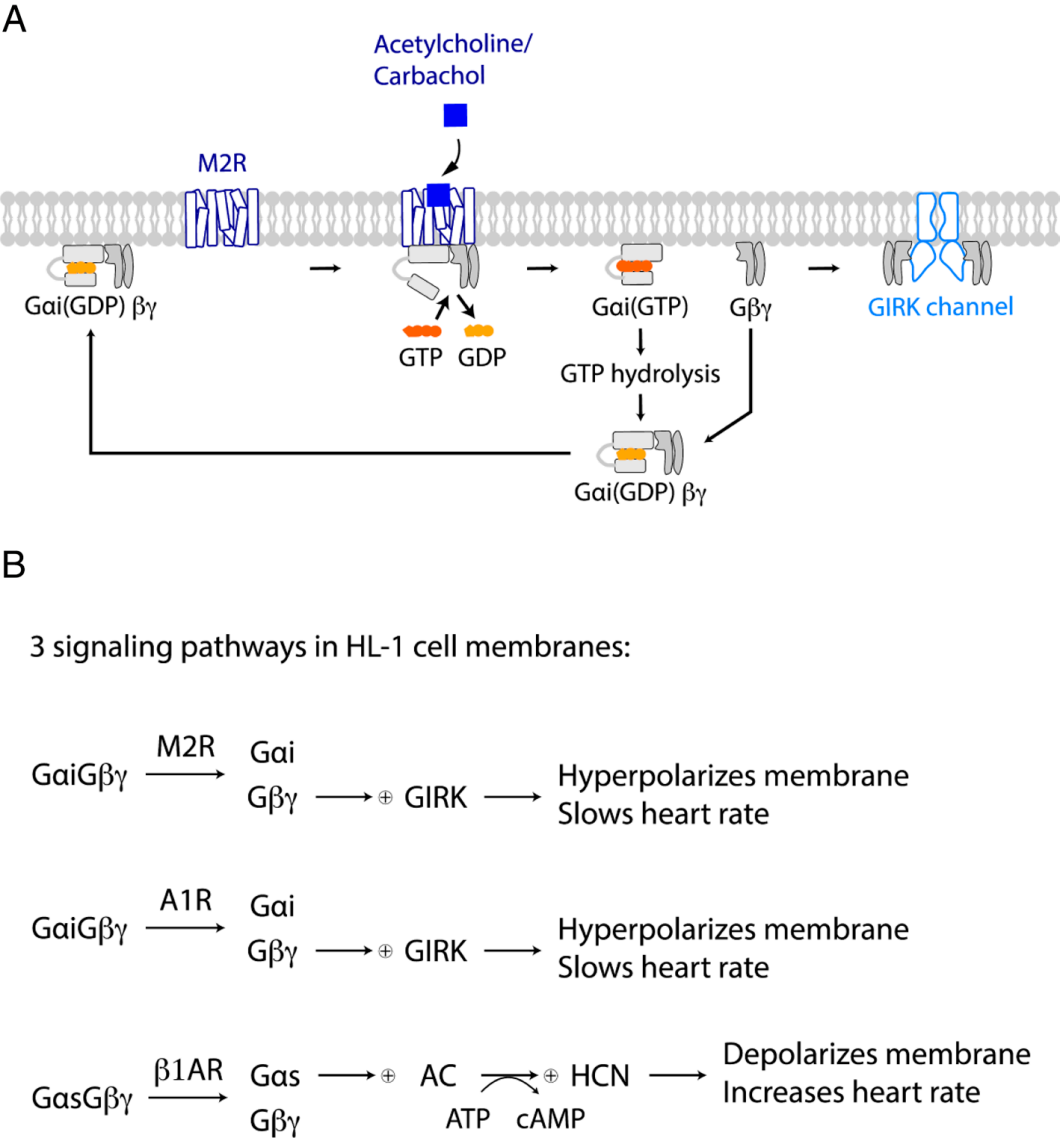
Coexisting signaling pathways in the HL-1 cell plasma membrane. (*A*) Schematic of the M2R-GIRK channel signaling pathway in HL-1 cell membranes. (*B*) M2R, A1R, and β1AR pathways in HL-1 cell membranes. Both M2R and A1R activate GIRK by releasing free Gβγ and thus hyperpolarize the membrane, which slows heart rate. Gαs(GTP) generated by β1AR activates AC, which synthesizes cAMP. cAMP facilitates HCN channel opening, which depolarizes the membrane and increases heart rate.

We focus primarily on the muscarinic type 2 G-protein-coupled acetylcholine receptor (M2R)-G-protein gated inward rectifier K^+^ (GIRK) channel signaling pathway, in which M2Rs open GIRK channels by providing Gβγ subunits, as depicted (Fig. 1*A*) (2–8). Our basic strategy is to measure the locations of M2Rs and GIRK channels in HL-1 cell membranes, calculate theoretical levels of channel activity from these distributions, and compare the calculations to electrophysiological measurements in HL-1 cells.

The M2R-GIRK channel pathway coexists in the same membranes with other signaling pathways (Fig. 1*B*). Adenosine receptors (A1Rs), like M2Rs, open GIRK channels by providing Gβγ subunits. Beta-adrenergic receptors (β1ARs) activate AC by providing Gαs subunits (9, 10). M2R and A1R both slow heart rate; β1AR does the opposite (11–15). As a secondary aim of this study, we label some proteins from these different pathways to search for patterns of connectivity that might correlate with functional output. Our findings lead us to put forth the hypothesis of dynamic connectivity in membrane protein signaling that depends on HOTS as an essential element.

## Results

### Positive Spatial Bias among Functionally Connected Proteins.

**Fig. 2.**
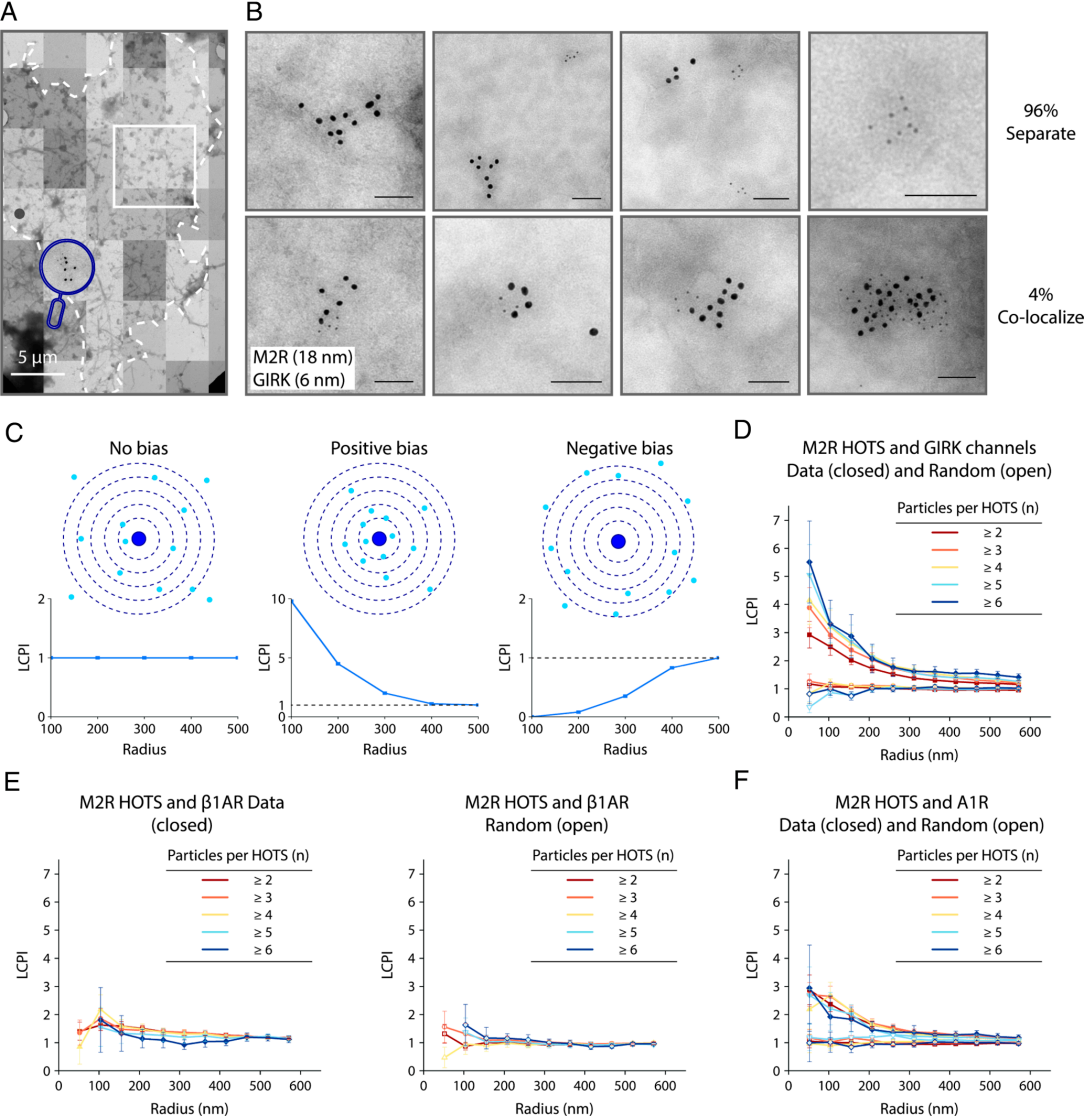
Detection of a spatial bias between membrane protein HOTS. (*A*) A representative M2R (18 nm) and GIRK (6 nm) double-labeled electron microscope montage of an unroofed HL-1 cell. The dashed perimeter outlines the boundary of the membrane. An example of an M2R-GIRK cocluster is shown in the magnifying glass. The region in the white square is addressed in Fig. 4. (Scale bars: 5 μm.) (*B*) Representative negative stain electron micrographs with double-labeled M2R (18 nm) and GIRK (6 nm) in HL-1 cells. *Top* row: Example of M2R and GIRK HOTS that do not colocalize. *Bottom* row: Example of M2R-GIRK coincident HOTS. Only 4% of GIRK channels colocalize with M2R HOTS (i.e., when the distance between the GIRK channel and M2R cluster centroids is less than 150 nm.). (Scale bars: 100 nm.) (*C*) Schematic of the Landmark Correlated Particle Index (LCPI) analysis used in panels (*D*–*F*) (16). LCPI = (fraction of total protein inside a circle)/(fraction of unroofed membrane area inside a circle). Dark blue central filled circles represent M2R HOTS centroids. Light blue dots represent other proteins. LCPI as a function of circle radius generated in each case is shown in the *Lower* panel. (*D*) LCPI analysis for M2R HOTS and GIRK channels (closed symbols) or randomly distributed GIRK channels (open symbols) in HL-1 cells. Different colors correspond to different M2R HOTS sizes. (In the randomized case, an equal number of GIRK channels are randomly distributed in the unroofed membrane.) Symbols show mean and SE from 17 electron microscope montages. (*E*) LCPI analysis as in panel (*D*) for M2R HOTS and β1AR (*Left*) or randomly distributed β1AR (*Right*) in HL-1 cells. Symbols show means and SE from 10 electron microscope montages. (*F*) LCPI analysis as in panel (*D*) for M2R HOTS and A1R (closed symbols) or randomly distributed A1R (open symbols) in HL-1 cells. Symbols show mean and SE from 13 electron microscope montages.

We prepared unroofed HL-1 cell membranes and immunogold labeled membrane proteins on electron microscope (EM) grids as described in the accompanying paper (1). Individual micrographs were stitched together to form a montage (Fig. 2*A*). In this montage, M2Rs were labeled with 18 nm gold particles, GIRK channels with 6 nm gold particles, and the coordinates of both were identified after pretraining the machine learning algorithm implemented in Dragonfly (Object Research Systems Inc.). The magnifying glass icon in Fig. 2*A* highlights an M2R HOTS adjacent to a GIRK channel HOTS.

We first examine whether M2R and GIRK channel HOTS are distributed on the membrane independently. Close inspection shows that most M2R and GIRK channel HOTS are isolated by themselves in the montage (Fig. 2 *B*, *Top* row), but some of them are very close and sometimes coincident (Fig. 2 *B*, *Bottom* row). To know whether coincident HOTS are by chance or whether there is a bias favoring their proximity, concentric circles were drawn around the centroid of each M2R HOTS and the relative density of encompassed GIRK channels was calculated as a function of the circle radius (16). Fig. 2*C* illustrates how this analysis detects a positive or negative bias, and Fig. 2*D* shows that a positive bias exists for M2R and GIRK channels to be near each other, with larger M2R HOTS exhibiting a stronger bias. We showed in an accompanying paper that all five membrane proteins form HOTS that are randomly distributed on the membrane (1). Here, we see that M2R and GIRK channel HOTS, while randomly distributed on the membrane, exhibit a relative bias such that they tend to be near each other.

We wondered whether some systematic error might make all pairs of membrane proteins appear to exhibit a positive bias. This is clearly not the case, as shown by the absence of a bias in the relative distributions of M2R and β1AR HOTS (Fig. 2*E*). M2R and A1R on the other hand exhibit a positive relative bias, weaker than that for M2R and GIRK channels, but clearly above a zero-bias determined after the positions of proteins are randomized computationally (Fig. 2*F*). At present, the colabeling experiments are limited by our small set of primary and secondary antibodies. But even with this small dataset, it seems that a pattern emerges. M2R and GIRK channels are physically connected by a positive bias in the separation of their HOTS, as are M2R and A1R. M2R and A1R both are Gαi coupled GPCRs that regulate similar downstream processes (10, 14, 15, 17). β1AR on the other hand, whose HOTS exhibit no bias with respect to M2R, is a Gαs coupled GPCR that regulates distinct, functionally opposing, downstream processes (10, 13).

### M2R HOTS as Localized Domains of Signal Transmission.

**Fig. 3.**
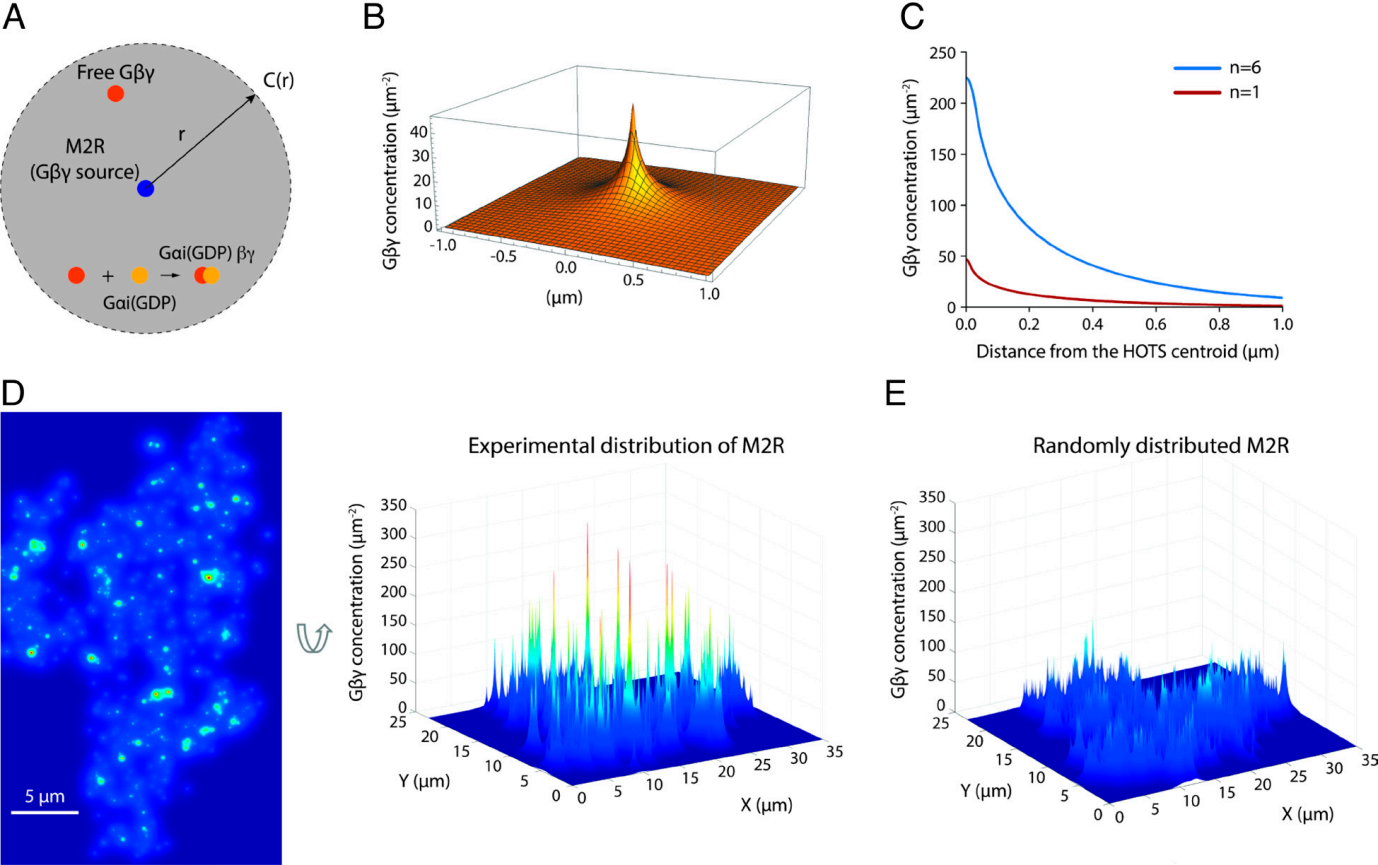
Calculating the Gβγ field in plasma membrane sheets. (*A*) Depiction of the system for solving the steady-state diffusion equation. The central blue circle represents an M2R source. G-protein trimers are assumed present at a constant concentration. Free Gβγ (red circle) [and Gαi(GTP), not shown] generated at the source diffuses until it recombines with Gαi(GDP) (yellow circle), the hydrolysis product of Gαi(GTP). The diffusion equation, $\nabla \cdot D\nabla C\left(r\right)-k\;C\left(r\right)=0,$ in which *D* and *k* are the diffusion coefficient and disappearance rate constant for Gβγ, is solved for the concentration of Gβγ at distance r subject to a near gradient boundary condition on a circular perimeter near the source: ∇Gβγ satisfies $\oint D_{G\beta \gamma }\nabla G\beta \gamma \;ds=\mathrm{rate}\;\mathrm{of}\;G\beta \gamma \;\mathrm{production}$ and a far boundary condition is set by Gβγ=0 at r=2.0 μm. We assume rate of $G\beta \gamma \;\text{production}=10\;s^{-1}$ (18), lifetime 2.0 s (20, 21), and diffusion coefficient 0.15 μm^2^/s (19). (*B*) Calculated steady-state 2-dimensional Gβγ concentration profile generated by a single M2R. Parameters are as described in panel (*A*). Gβγ concentration is high near M2R and decays with distance from the source. (*C*) Steady-state Gβγ concentration generated by a single M2R (red) and a HOTS containing 6 M2R (blue). (*D*) The Gβγ field generated from the M2R-GIRK double-labeled montage in Fig. 2*A*. The Gβγ field was calculated from the coordinates of M2R HOTS centroids and HOTS sizes using the parameters and equation described in panel (*A*), with source rate proportional to n, the number of M2Rs inside a HOTS. Color and the z coordinate indicate the concentration of free Gβγ generated by activated M2R. (Scale bars: 5 μm.) (*E*) The hypothetical Gβγ field generated for the montage in Fig. 2*A* after M2R is randomly redistributed over the membrane.

Focusing on HL-1 cell montages that show the locations of both M2R and GIRK channel labels, we next estimate through calculation the level of GIRK channel activity that should in theory be elicited through M2R stimulation. In a first step, we calculate a hypothetical steady-state Gβγ concentration (number of proteins per area in units $\mu m^{-2}$) as a function of position on the cytoplasmic surface of the membrane. We call this concentration as a function of position the Gβγ field. To see how this calculation is made, first imagine that a single active M2R serves as a source of Gβγ subunits generated at a known rate (Fig. 3*A*) (18, 19). Once released from M2R, the Gβγ subunits undergo Brownian motion with a characteristic diffusion coefficient (19), while existing for a characteristic lifetime before disappearing through sequestration by Gαi-GDP (20, 21). The Gβγ field is obtained by solving the steady-state diffusion equation $\nabla \cdot D\nabla C\left(r\right)-k\;C\left(r\right)=0$ for the concentration of Gβγ, $C\left(r\right)$, at distance r from the source. D and k are the diffusion coefficient and disappearance rate constant ($\tau_{\mathrm{lifetime}}^{-1}$) for Gβγ, respectively, and the source rate is introduced through a gradient boundary condition at the source perimeter (*Materials and Methods* and Fig. 3*A*). The Gβγ field surrounding a single M2R is depicted in Fig. 3 *B* and *C* (red curve) and surrounding a HOTS containing six M2Rs in Fig. 3*C* (blue curve). Fig. 3*D* shows the Gβγ field for the entire montage in Fig. 2*A*. It contains punctate regions of high Gβγ concentration compared to a membrane with an equal number of randomly distributed M2Rs (Fig. 3*E*). While the Gβγ field may be inaccurate because it is based on uncertain values for the generation rate and lifetime, it captures the concept that M2R HOTS will produce concentration gradients of Gβγ on the membrane, with high concentrations near larger M2R HOTS.

**Fig. 4.**
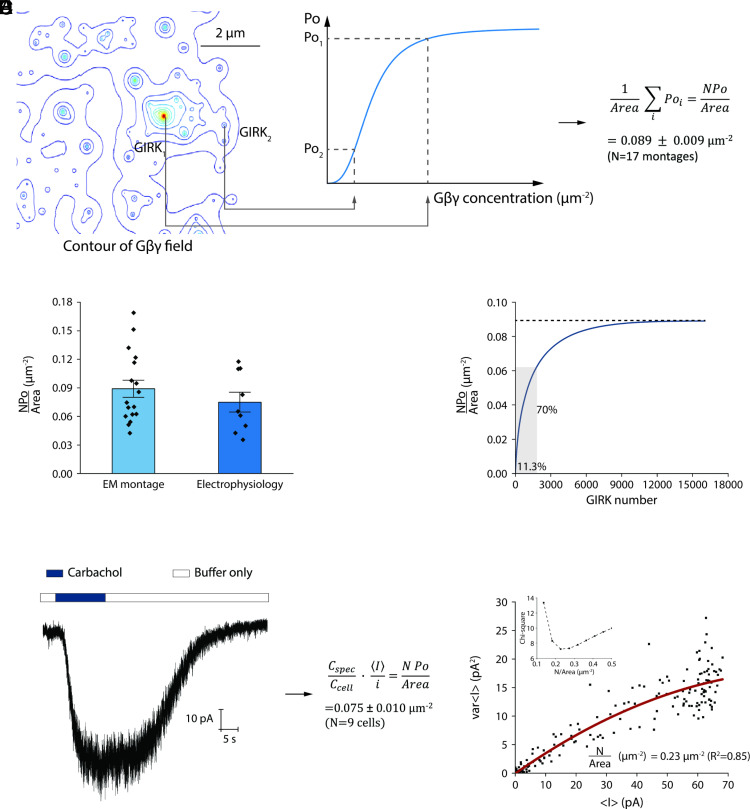
Comparing calculated open probability densities from structure maps to electrophysiological measurements. (*A*) Schematic showing the procedure to predict *NPo*/μm^2^ from the Gβγ field. The Gβγ concentration contour plot with 15 levels generated for the square region in Fig. 2*A* is shown (*Left*). GIRK channels in the M2R-GIRK double-labeled electron micrographs are assigned a Gβγ concentration based on their position in the Gβγ field. The open probability (${\mathrm{Po}}_{i}$) of each (ith channel) in the field was calculated from the concentration dependence of GIRK channel activation (*Right*), summed, and divided by the measured membrane area to estimate *NPo*/*Area* (*Materials and Methods*). (*B*) Open probability density, *NPo*/*Area* ($\mu m^{-2}$), calculated from electron micrograph montages from HL-1 cells as described in the text and in panel (*A*). Symbols show individual outcomes from 17 montages (symbols) with bar height and error bars showing the mean and SE (0.089 ± 0.009 μm^−2^) from the 17 montages (*Left*). Open probability density, *NPo*/*Area* (μm^−2^), calculated from electrophysiological current and membrane capacitance measurements on HL-1 cells as shown below. Symbols show outcomes from nine cells (symbols) with bar height and error bars showing the mean and SE (0.075 ± 0.010 μm^−2^) from the nine cells (*Right*). (*C*) Cumulative open probability density plot generated from electron micrograph montages. Channels from 17 montages were ordered in a list according to their calculated open probability, from highest to lowest. A graph of the cumulative sum of open probabilities in the list (divided by the total montage area) shows that most of the open probability density comes from a small fraction of the total GIRK channels. (*D*) A representative whole-cell trace showing the current response of GIRK channels to M2R activation by carbachol and expression to calculate *NPo*/*Area*. Carbachol was applied at 10 μM and then removed by perfusion. Voltage was held at –60 mV. Buffer conditions are described in *Materials and Methods*. The mean and SE from nine current traces are shown. C_cell_ is the measured cell capacitance and C_spec_ = 0.009 pF/μm². (*E*) Current variance, $var\left\langle I\right\rangle$, and mean current, $\left\langle I\right\rangle$, were measured from traces like those in panel (*D*). In the graph, $var\left\langle I\right\rangle$ is plotted as a function of $\left\langle I\right\rangle$ from the trace in (*D*) (symbols). The current signal was low pass filtered at 1 kHz and digitized at 10 kHz. $var\left\langle I\right\rangle$ and $\left\langle I\right\rangle$ are calculated for every 3,000 nonoverlapping data points. The red curve corresponds to the function $var\left\langle I\right\rangle =i\left\langle I\right\rangle -\frac{{\left\langle I\right\rangle }^{2}}{N}$ (24). The single channel current i (~1.0pA) was measured independently and therefore the function contains a single free parameter, N, the number of channels activated by carbachol application. Capacitance measurements on the same cell permit calculation of membrane area. N divided by the membrane area gives the density of activated channels, *N*/*Area* (μm^−2^). The chi-square plot (*Inset*) for this cell shows a minimum value for *N*/*Area* ~0.23 μm^−2^. The mean and SE of measurements in nine cells is 0.32 ± 0.04 μm^−2^.

In a second step, we use the Gβγ field to assign a local Gβγ concentration to each GIRK channel in the montage (Fig. 4*A*). Then, using the relationship between GIRK channel activity and Gβγ concentration (22) (*Materials and Methods*) we assign an open probability ${\mathrm{Po}}_{i}$ to each channel i, sum over all channels, and divide by the cell membrane area as outlined (Fig. 4*A*). This yields a calculated open probability density (*NPo*/*Area*) for *N* channels with mean open probability Po in a membrane area Area. Repeating this procedure for 17 montages from HL-1 cells yields a calculated mean open probability density of $0.089\pm 0.009\;\mu m^{-2}$ (Fig. 4 *A* and *B*). The density of GIRK channels in these same montages is $2.82\pm 0.26\;\mu m^{-2}$, about 30 times the open probability density. From this, we conclude that Po is about 0.03. While this value is only approximate, it clearly indicates that the mean open probability of GIRK channels in HL-1 cells is small. The reason for this becomes clear if we take all GIRK channels from the 17 montages, order them in a list of ${\mathrm{Po}}_{i}$ from highest to lowest, and graph the cumulative open probability divided by the membrane area (Fig. 4*C*). Around 10% of the channels account for nearly 70% of the open probability density because only those channels near large M2R HOTS tend to open. Thus, according to this calculation, signaling is predicted to occur at large M2R HOTS.

### Electrophysiological Assessment of Localized Signaling.

We next examine whether electrophysiological measurements are consistent with the above prediction. We used whole-cell patch recording with HL-1 cells to measure GIRK currents while maximally stimulating M2Rs with the agonist carbachol (Fig. 4*D*). From the relationship $\left\langle I\right\rangle =iNPo$, which relates mean current $\left\langle I\right\rangle$ to single channel current i (∼1.0 pA under our recording conditions), we calculate $NPo=\frac{\left\langle I\right\rangle }{i}$ from the two measured quantities $\left\langle I\right\rangle$ and i. For each HL-1 cell, we also measured the membrane capacitance to estimate the cell membrane area from the constant $0.009\;\mathrm{pF}/\mu m^{2}$ for cell membranes (23). From nine cells, the experimental open probability density at maximal channel activation is $0.075\pm 0.010\;\mu m^{-2}$ (Fig. 4*B*). This value is close to the calculated value using montages, $0.089\pm 0.009\;\mu m^{-2}$, but this correspondence does not necessarily mean the electrophysiology experiments agree with the calculation from the montages. The important outcome of the montage calculation was that the overall open probability is low because only a relatively small fraction of the channels open (in contrast to all channels opening to a lesser degree) (Fig. 4*C*). Thus, we need to tease apart open probability density (*NPo*/*Area*) into channel density (*N*/*Area*) and open probability (Po) in the electrophysiological recordings. This can be done by analyzing current fluctuations in the electrophysiological recordings (24).

In Fig. 4*D*, the current fluctuations are larger near the maximum current when carbachol is applied and smaller as the current decreases after carbachol is removed. For a population of channels that are identical and gate independently, the current mean and variance are given by $\left\langle I\right\rangle =iNPo$ and $var\left\langle I\right\rangle =i^{2}Po\left(1-Po\right)$, respectively. Combining these expressions gives $var<I>=i<I>-\frac{{<I>}^{2}}{N}$ (24). The graph in Fig. 4*E* shows $var\left\langle I\right\rangle$ as a function of $\left\langle I\right\rangle$ for the HL-1 cell current trace in Fig. 4*D*. This analysis, carried out in nine cells, together with capacitance measurements, gives mean values for channel density NArea=0.32±0.04μm-2 and maximum open probability Po=0.27±0.06. In words, this analysis indicates that the population of GIRK channels in HL-1 cells that respond to M2R stimulation has a density of $0.32\;\mu m^{-2}$ and mean maximal open probability of 0.27. Notably, this channel density is about one-tenth the number observed in HL-1 cells using gold labels ($2.82\pm 0.26\;\mu m^{-2}$ ) and the open probability about ten times that estimated in the above montage-based calculation (~0.03). Thus, the electrophysiological measurements are consistent with the conclusion of montage calculations, that only a fraction of GIRK channels are activated upon M2R stimulation. The critical reader will note that the fraction (~0.1) of channels that are activated will likely experience Gβγ gradients and therefore not gate identically, in contradiction to the assumption in our analysis of identically gating channels. To this criticism, we could state our conclusion as follows. If all the GIRK channels present in the membrane were equally activated, then the analysis of channel fluctuations should have yielded a channel density near 3.0 $\mu m^{-2}$ and an open probability near 0.03. Instead, the analysis points to a smaller population of channels opening to a greater extent. Presumably, the subset of activatable channels are those near larger M2R HOTS.

The distribution of GIRK channels in the Gβγ field provides a natural and simple explanation for why only a relatively small fraction of GIRK channels open, but it is not an exclusive explanation. It could be that a large fraction of the GIRK channels is silent for another reason. PIP2 is an essential cofactor, but it seems unlikely to be deficient (25). GIRK channels are also regulated by other mechanisms (26). While the correlation between the structural mapping and electrophysiology is good, our analysis assumes that the GIRK channels are activatable if the Gβγ concentration is sufficient.

### Calculating the Effect of HOTS and Their Spatial Bias on Signaling.

**Fig. 5.**
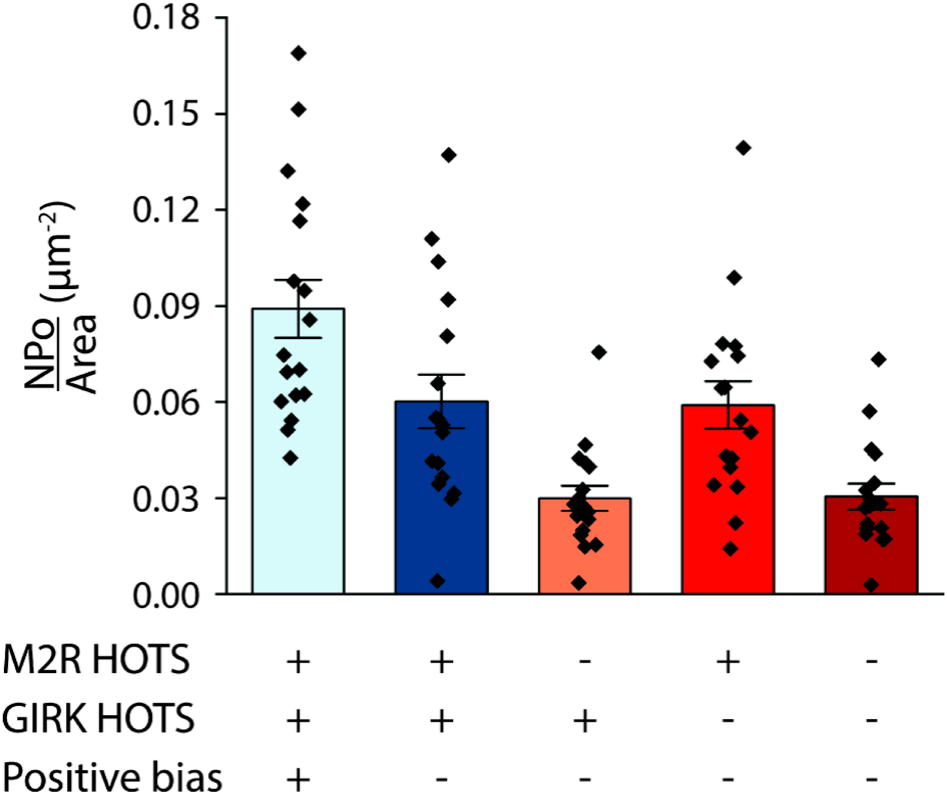
The calculated effect of HOTS and spatial bias on open probability density. Open probability density, *NPo*/*Area* (μm^−2^), calculated from electron micrograph montages from HL-1 cells as shown in Fig. 4*B* (light blue). *NPo*/*Area* (μm^−2^) calculated from hypothetical Gβγ fields generated from the montages after the positive bias is removed (dark blue). Positive bias is the bias between M2R HOTS and GIRK channels. To remove the positive bias, the positions of M2R HOTS were randomized on the membrane without changing the HOTS size distribution. *NPo*/*Area* (μm^−2^) was calculated from hypothetical Gβγ fields generated from montages after M2R (orange) or GIRK (red) or both proteins (dark red) were randomly redistributed on the membrane. Symbols show individual outcomes from 17 montages with bar height and error bars showing the mean and SE (0.089 ± 0.009 μm^−2^, 0.060 ± 0.008 μm^−2^, 0.030 ± 0.004 μm^−2^, 0.059 ± 0.007 μm^−2^, and 0.030 ± 0.004 μm^−2^, respectively, from *Left* to *Right*) from the 17 montages.

We next examine through calculation what effect HOTS and spatial bias would have on signaling if M2R and GIRK channels measured in the montages are redistributed computationally. For each montage, redistributions are applied, Gβγ fields calculated, and open probability densities determined as described above (Fig. 5). In all cases, the number of channels and receptors is kept constant. By randomly distributing M2R or GIRK channel HOTS, their oligomeric states are preserved but the positive spatial bias between them is removed. This reduces the open probability density. When M2Rs are randomly distributed so their HOTS disappear, while maintaining GIRK channel HOTS, the open probability density decreases further. Note that because the spatial bias is defined using the centroids of HOTS, we cannot dissolve HOTS and maintain the bias (without a model, see below). When GIRK channels are randomly distributed but M2R HOTS maintained, the open probability density is, within error, equal to removal of the spatial bias alone. Finally, when both proteins are randomly distributed the open probability density equals that when M2Rs are randomly distributed. Taken together, these calculations suggest that M2R HOTS are very important to signaling and that the spatial bias strengthens the signal. GIRK channel HOTS appear unimportant; however, as we will see below, they may play a role in creating the spatial bias.

### Simulating the M2R-GIRK Pathway.

**Fig. 6.**
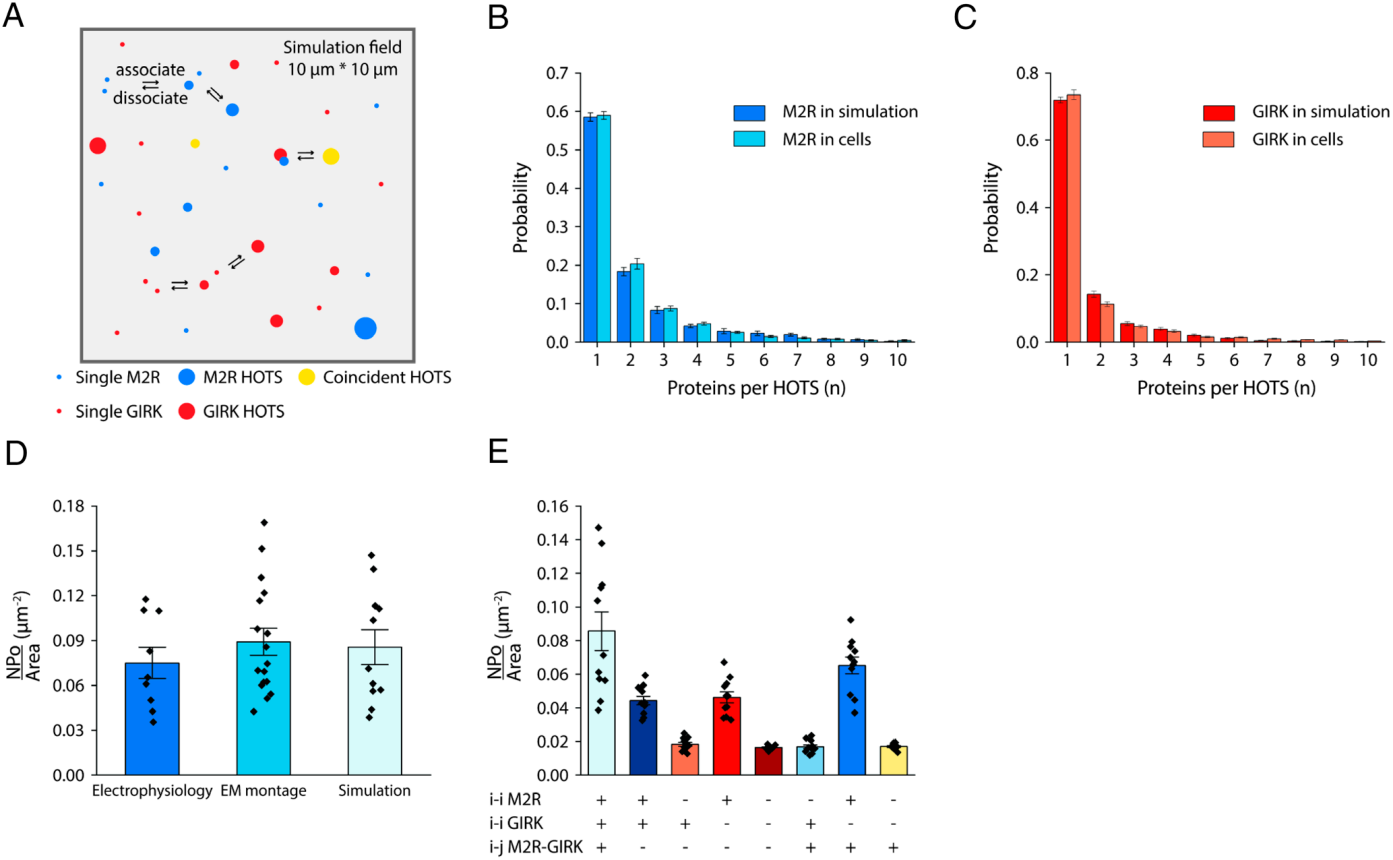
Simulating the M2R-GIRK pathway to assess the role of *i-i* and *i-j* interactions. (*A*) Schematic of the simulation with blue, red, and yellow circles representing M2R, GIRK channel, and coincident HOTS; circle size proportional to the number of proteins in a HOTS or coincident HOTS. In the simulation, the total M2R density is 2 μm^−2^ and total GIRK density 3 μm^−2^, near those measured in HL-1 cells (1.94 ± 0.13 μm^−2^ for M2R and 2.82 ± 0.26 μm^−2^ for GIRK using 18 nm and 6 nm gold labels, respectively). In the first frame of the simulation, particles representing proteins were distributed randomly. Particles diffuse with a diffusion coefficient of 0.1 μm^2^/s. M2R and GIRK can each self-assemble reversibly to form HOTS and with each other to form coincident HOTS. Details of the diffusion process and conditions under which an oligomerization reaction occurs between nearby proteins is described in *Materials and Methods*. Particle distributions were first analyzed after 20,000 simulation steps and then analyzed every 5,000 steps. (*B* and *C*) Normalized (meaning sum of probabilities equals 1.0) M2R or GIRK HOTS size distributions in HL-1 cell double-labeled montages or in the simulation. Data represent means and SE from 17 electron micrograph montages or 11 independent simulations. (*D*) Open probability density, *NPo*/*Area* (μm^−2^), calculated from electrophysiological current or electron micrograph montages from HL-1 cells as in Fig. 4*B*. Symbols show individual outcomes from nine cells (symbols) or 17 montages (symbols) with bar height and error bars showing the mean and SE (0.075 ± 0.010 μm^−2^ and 0.089 ± 0.009 μm^−2^) from the nine cells (*Left*) and 17 montages (*Middle*). Open probability density, *NPo*/*Area* (μm^−2^), calculated from simulations. The Gβγ field was calculated for each simulation as described in Fig. 3*D*. Open probability density was then calculated as described in Fig. 4*A*. Symbols show individual outcomes from 11 simulations (symbols) with bar height and error bars showing the mean and SE (0.086 ± 0.012 μm^−2^) from the 11 simulations (*Right*). (*E*) Open probability density, *NPo*/*Area* (μm^−2^) calculated from simulations at different conditions. *i-i* refers to self (i.e., M2R-M2R and GIRK-GIRK) interactions and *i-j* refers to nonself (i.e., M2R-GIRK) interactions. To remove *i-i* M2R, *i-i* GIRK or *i-j* M2R-GIRK interactions, the corresponding association probability (*Materials and Methods*) was set to zero. The Gβγ field was calculated for each simulation, and open probability density then calculated from the Gβγ field. Symbols show individual outcomes from 11 simulations with bar height and error bars showing the mean and SE (0.086 ± 0.012 μm^−2^, 0.044 ± 0.003 μm^−2^, 0.018± 0.001 μm^−2^, 0.046 ± 0.003 μm^−2^, 0.016 ± 0.0004 μm^−2^, 0.017 ± 0.001 μm^−2^, 0.065 ± 0.005 μm^−2^ and 0.017 ± 0.001 μm^−2^, respectively, from *Left* to *Right*) from the 11 simulations.

The physical basis of HOTS formation appears to be favorable short-range, specific interactions between like proteins (1). We refer to these as *i-i* interactions: M2R assembles with itself through its own *i-i* interactions, and GIRK with itself through its own *i-i* interactions. We hypothesize that protein–protein interactions also account for the positive bias between M2R and GIRK channel HOTS and call these *i-j* interactions. The *i-j* interactions would be very weak, just enough to create the positive bias. We ask, can we predict the organization of proteins observed in cell membranes and the response of the M2R-GIRK signaling pathway knowing only the protein concentrations, the two *i-i* interactions and the one *i-j* interaction? We applied a diffusion coefficient so that the system could evolve from initial random configurations. We coded the *i-i* and *i-j* interactions by assigning association and dissociation probabilities that are functions of rate constants, as described in *Materials and Methods*. These probabilities contain geometric terms that encode information about HOTS size. Each reaction diffusion simulation was run long enough to approximate a stationary configuration (*SI Appendix*, Fig. S2). A cartoon depicts the process with blue, red, and yellow circles corresponding to M2R, GIRK, and coincident HOTS; circle size being proportional to the number of proteins in a HOTS or coincident HOTS (Fig. 6*A*). In Movie S1, we show a movie of this dynamic process. With appropriate *i-i* and *i-j* interactions, HOTS size distributions mimic the experiment (Fig. 6 *B* and *C*). Because the simulation can approximate the experimentally observed organization of these proteins in the membrane, it also yields similar open probability densities (Fig. 6*D*).

We examined the effect of modifying the *i-i* and *i-j* interactions to ask which elements of this mechanism are important for efficient signal communication. Eliminating the bias by removing the *i-j* interaction reduced the open probability density (Fig. 6*E*). Eliminating the *i-i* interactions for M2R and GIRK individually or both together, while at the same time eliminating the *i-j* interaction, also reduced the open probability density. These outcomes are consistent with those obtained with direct computational randomization of montage coordinates (compare Figs. 5 and 6*E*).

Beyond showing that agreement with experiments could be achieved, the simulations let us interrogate a mechanistic point not addressable through modification of the montage data because when randomizing aspects of a montage it is not possible to eliminate HOTS without eliminating the spatial bias. By manipulating *i-i* and *i-j* interactions independently in simulations, we can address, albeit in a model-dependent manner, their individual contributions to signaling. When we run these simulations, we observe an intriguing result. The *i-j* interaction increases the K^+^ current magnitude to the greatest extent only when M2R and GIRK channel HOTS are present simultaneously (Fig. 6*E*). This is because the HOTS, through their multivalency, supply an avidity boost to the weaker *i-j* interaction. This might explain why the GIRK channel forms HOTS. It is easy to understand why M2R HOTS are beneficial—because localized domains of high Gβγ concentration are required to activate GIRK channels. The rationale for GIRK channel HOTS (and a second rationale for M2R HOTS), to enhance the *i-j* interaction, is more subtle, but also can promote efficient signaling.

In a previous study, we proposed a kinetic explanation for the observation that β1AR stimulation in cardiac pacemaker cells does not activate GIRK channels even though β1AR generates Gβγ (2). In that study, using a bioluminescence resonance energy transfer (BRET) assay we found a significantly lower rate of Gβγ generation by β1AR compared to M2R. To model that process with the simulation developed here, we entered the experimental β1AR concentration [about half the M2R concentration (1)] and a Gβγ generation rate one-tenth that of M2R. The simulation predicts essentially no activation of GIRK channels by β1AR (*SI Appendix*, Fig. S1). Therefore, the simulation is compatible with the known specificity of the M2R-GIRK channel signaling pathway.

## Discussion

This study presents two kinds of data, structural and electrophysiological. The structural data are the coordinates of membrane proteins on HL-1 cell membranes, measured by unroofing the cells, labeling the proteins, and mapping their positions on the membrane with an electron microscope. They show that the membrane proteins form small clusters called HOTS, described in the preceding paper, and that HOTS of certain protein pairs, such as M2R and GIRK channels, are biased to be nearer each other than predicted by chance. The electrophysiological data are ionic and capacitive currents recorded from HL-1 cells. They show that the current density is small compared to the known density of channels in the membrane and that this is mainly because only a fraction of the GIRK channels open, not because all channels open to a small extent.

To examine whether the structural and electrophysiological data are congruous, we use the structural data to calculate a theoretical level of GIRK channel activation. We first calculate a Gβγ field from a steady-state diffusion equation, which requires three values: a diffusion coefficient, production rate, and lifetime of Gβγ. The diffusion coefficient for Gβγ is ~0.15 μm^2^/s (19). The rate of Gβγ production by M2R (1.0 to 10.0 s^−1^) (18, 19) and lifetime ~2.0 s (20, 21) have greater uncertainties and can be influenced by multiple factors in cells (27). Despite these uncertainties, the calculation predicts gradients of Gβγ concentration over a wide range of input values, with higher concentrations near larger HOTS. To convert the Gβγ field to open probability density (proportional to current density in electrophysiology experiments), we use the structural data (i.e., the mapping) to locate GIRK channels in the field and assign a Gβγ concentration to each, with which we calculate an open probability for every GIRK channel in the membrane (22) (*Materials and Methods*). The basic conclusion we reach is this: GIRK channels near large M2R HOTS open more because the Gβγ concentration is higher. This conclusion aligns with the electrophysiology data, which show that only a fraction of GIRK channels, presumably those near larger M2R HOTS, open upon maximal stimulation of M2R with carbachol. We note that submaximal stimulation of M2R, likely to occur in a physiological setting, will probably restrict M2R to GIRK signaling even more severely to larger HOTS.

The first study in this series indicated that interactions between neighboring like proteins in HOTS are mediated by direct protein contacts, which here we call *i-i* interactions (1). We propose that specific interactions between unlike proteins, for example, M2R and GIRK, can also occur; we call these *i-j* interactions. Our simulations of the M2R-GIRK channel pathway show that *i-i* interactions can create HOTS of each protein, and *i-j* interactions can bring different HOTS together, i.e., create a positive spatial bias. From the simulation, we learn that if the *i-j* interactions are very weak, the multivalent nature of HOTS is required to bring them together. Thus, we propose that GIRK channels form HOTS to promote their proximity toward M2R HOTS.

The M2R-GIRK signaling pathway has been studied for many years. It has been proposed that M2Rs and GIRK channels form a static macromolecular complex in the membrane (28–32); however, we have been unable to isolate such a complex. In this study, we propose a modification of this view. M2R and GIRK channels each form HOTS through weak *i-i* interactions. The M2R HOTS transiently create local regions of high Gβγ concentration. GIRK channels in the vicinity of the larger HOTS can thus be activated. Weak *i-j* interactions, enhanced by the multivalent nature of M2R and GIRK channel HOTS, increase the probability that GIRK channel HOTS will be near M2R HOTS. In contrast to the connectivity between components of an electronic circuit, or a signaling pathway based on static protein complexes, the connectivity we propose is dynamic. HOTS are the signature feature of self-assembly in the weak interaction regime. In the simulation, and we propose in cells, HOTS underlie dynamic connectivity to produce efficient signaling.

There are distinct advantages to signaling with dynamic connectivity. First, components such as G-proteins can be shared among pathways. Component sharing might be related to the positive spatial bias that we observe between M2R and A1R HOTS (Fig. 2*F*), which share the same Gαi and Gβγ proteins (10, 14, 17). Second, dynamic connectivity would also permit rapid regulation owing to the transience of the underlying structures, giving rise to switch-like on–off behavior. And third, transience goes together with plasticity. Phosphorylation of a component, for example, could rapidly alter the connectivity in a signaling pathway by altering *i-i* interactions to mediate the formation or dissolution of HOTS, or *i-j* interactions to modify a spatial bias between two components of the signaling pathway.

While the present study focuses on the role of HOTS in the M2R-GIRK channel signaling pathway, we showed in the preceding paper that five different membrane proteins form HOTS. We suspect that other signaling pathways might also employ similar principles of self-assembly to create localized regions where signaling occurs, with dynamic connectivity among protein components that comprise the pathway. Consider, for example, high-conductance Ca^2+^-activated K^+^ (BK) channels, which localize near Cav1.3 voltage-dependent Ca^2+^ channels in some neurons, permitting the two ion channels to work together as a unit to regulate each other’s activity (33). Localization microscopy showed that clusters of Cav1.3 channels surround clusters of BK channels. While the mechanism of clustering and positive bias between clusters in this case is unknown, the picture is easily explicable if BK and Cav1.3 channels each form their own HOTS, and perhaps bulk phase clusters, through *i-i* interactions, and then exhibit a positive bias through *i-j* interactions. In another example, different Gα_s_ coupled GPCRs in cell membranes have been shown to create their own compartmentalized domains, many tens of nanometers in size, where cAMP levels are locally high (34). This way different Gα_s_ coupled receptors do not occlude each other’s response. In a third example, PIP2 depletion in localized regions of a cell membrane near Gα_q_ coupled GPCRs is thought to underlie receptor specificity in the regulation of PIP2-dependent ion channels (35).

It might seem that HOTS would be useful only for mediating nonsynaptic humoral signaling, where the receptors and channels are distributed across the cell membrane. But consider the following: When sympathetic neurons form synapses with heart cells, they appear to recruit and enrich β1ARs at the postsynaptic membrane (36). Similarly, denervation of skeletal muscles causes the nicotinic acetylcholine receptor ion channels (nAChRs), which are normally concentrated at the synapse, to spread out across the membrane (37, 38). In these examples, the synapse appears to serve as an attractor, presumably through *i-j* interactions between the receptor or channel and a synaptic protein. Thus, one might imagine if the receptor or channel spontaneously forms HOTS through its own *i-i* interactions, then it would be more readily recruitable to the synapse owing to the multivalency of a HOTS. Indeed, it appears that nAChRs do form clusters outside the synapse (37, 38). It will be interesting to see whether other synaptic receptors and channels form HOTS or bulk phase clusters (1) when they exist outside the synapse.

We chose the name higher-order transient structure because it emphasizes key features of these assemblies. “Higher-order” conveys the notion that a constituent protein unit—an ion channel, GPCR, enzyme—is functional by itself, but forms a higher-order assembly through specific self-recognition. “Transient” conveys the notion of weak interaction, which governs the size distribution of the assemblies and emphasizes the short timescale over which HOTS might be biologically important. “Structure” implies they are genetically selected supramolecular units. Our analysis of specific membrane proteins and their self (*i-i*) interactions have led us to a precise definition of HOTS, and how HOTS comprising different proteins can interact with each other through nonself (*i-j*) interactions at the membrane surface. We cannot help but wonder whether HOTS might also operate in other compartments of the cell, including the cytoplasm and nucleus, and whether they might deepen our understanding of biomolecular condensates, reviewed in ref. 39, and quinary interactions, reviewed in refs. 40 and 41.

In final summary, we hypothesize that HOTS mediate signaling on the cell membrane by spontaneously creating localized domains where second messengers are produced at higher concentrations than would be the case if the membrane proteins did not self-assemble and were thus randomly distributed. The HOTS are mediated by *i-i* interactions. A positive spatial bias is created by multivalent *i-j* interactions between HOTS of different protein species that have evolved to interact. In this hypothesis, the concentrations of signaling proteins and their set of *i-i* and *i-j* interactions should spontaneously give rise to the properties of multiple coexisting pathways through their dynamic connections.

## Materials and Methods

### Antibodies.

Antibodies used in this study were the same as in our companion paper (1).

### Cell Culture.

Cells were cultured as described in our companion paper (1).

### Unroofing, Immunolabeling, and Distribution Analysis.

The unroofing and immunolabeling procedures were performed as described in our companion paper (1).

To quantify the positional relationship between two proteins, EM micrographs were taken on a Tecnai G2 Spirit BioTWIN Transmission Electron Microscope (ThermoFisher) with a pixel size of 0.979 nm/pixel. EM micrographs were then stitched using SerialEM (42). The gold particle annotation was performed in Dragonfly version 2021.1 for Windows (Object Research Systems Inc.) as described in our companion paper (1), and coordinates of both proteins were exported separately. The M2R distribution was first analyzed in the software Gold In-and-Out to obtain coordinates of M2R cluster centroids and cluster sizes with a distance threshold of 55 pixels (16). The positional relationships between M2R clusters and other proteins (GIRK, β1AR, or A1R) were then analyzed using the Gold Rippler function in the Gold In-and-Out software (16). Random simulation was also performed using the Gold In-and-Out software.

### Electrophysiology and Analysis.

The electrophysiology experiments were performed as described in our companion paper with minor modifications (1). Before the recording, HL-1 cells were kept in a bath solution of 10 mM HEPES-KOH pH 7.4, 120 mM NaCl, 20 mM KCl, 2 mM CaCl_2_, and 1 mM MgCl_2_. During the recording, the extracellular solution was changed to either the high K^+^ solution (10 mM HEPES-KOH pH 7.4, 80 mM NaCl, 60 mM KCl, 2 mM CaCl_2_, 1 mM MgCl_2_, 10 mM D-glucose) or high K^+^ solution supplemented with 10 μM carbachol or 2 μM tertiapin Q by a perfusion pencil placed close to the recorded cell. The current signal was low pass filtered at 1 kHz and digitized at 10 kHz.

For the nonstationary noise analysis described in Fig. 4, the current traces were exported from pClamp software (Molecular Devices). The mean current, $\left\langle I\right\rangle ,$ and current variance, $var\left\langle I\right\rangle ,$ were calculated for every 3,000 nonoverlapping data points (0.3 s intervals). The background current and variance were estimated from the current after the tertiapin Q application and subtracted. $var\left\langle I\right\rangle$ was then plotted as a function of $\left\langle I\right\rangle$. The plot was fitted with the quadratic $var\left\langle I\right\rangle =i\left\langle I\right\rangle -\frac{{\left\langle I\right\rangle }^{2}}{N}$ (24). The single GIRK channel current, *i* (~1.0 pA), was measured independently in outside-out patches excised from HL-1 cells. The voltage and buffer used in the outside-out patch experiments were the same as in the whole-cell experiments. Therefore, the quadratic function contains a single free parameter, N, the number of channels activated by carbachol application. Po is then calculated for any mean current $\left\langle I\right\rangle$ through the relationship $\left\langle I\right\rangle =iNPo$. The maximum Po is associated with the maximum mean current. Membrane area was calculated from the membrane capacitance, determined using a voltage ramp, with the specific membrane capacitance taken as $0.009\;\mathrm{pF}/\mu m^{2}$ (23). N divided by the membrane area yielded the density of GIRK channels that are activated by M2R.

To calculate the open probability density, *NPo*/*Area* for each cell, the maximum current after M2R activation was divided by the single GIRK channel current, i, and multiplied by the ratio of specific membrane capacitance to measured cell membrane capacitance.

### Calculation of the Gβγ Field and Open Probability Density *NPo*/*Area* from M2R-GIRK Double Labeled Montages.

To generate the Gβγ field, steady-state free Gβγ concentration profiles generated by M2R clusters of different sizes were first calculated using Mathematica [Wolfram Research, Inc., Mathematica, Version 14.1, Champaign, IL (2024)] scripts described previously (2). In the script, the diffusion equation, $\nabla \cdot D\nabla C\left(r\right)-k\;C\left(r\right)=0,$ in which *D* and *k* are the diffusion coefficient and disappearance rate constant for Gβγ, is solved for the concentration of Gβγ at distance r subject to a near gradient boundary condition on a circular perimeter near the source: ∇Gβγ satisfies $\oint D_{G\beta \gamma }\nabla G\beta \gamma \;ds=\mathrm{rate}\;\mathrm{of}\;G\beta \gamma \;\mathrm{production}$ and a far boundary condition is set by Gβγ=0 at r=2.0 μm. A Gβγ generation rate of 10 $s^{-1}$, a diffusion coefficient of 0.15 μm^2^/s, and a Gβγ lifetime of 2.0 s ($k=0.5\;s^{-1}$ ) were used (18–21). M2R HOTS centroid coordinates and HOTS sizes were then obtained from the distribution analysis described above. With these, we calculated a Gβγ field for every M2R-GIRK double-labeled montage using a MATLAB (MathWorks) script.

GIRK channels from the same double-labeled montage were mapped onto the Gβγ field using a MATLAB script. The free Gβγ concentration that each channel experiences under steady-state activation of M2R was assigned to a z-coordinate for each channel. The open probability, Po, was calculated using the expression (22)

$$Po=\frac{G\beta \gamma \;mole\;fraction^{n}}{K_{d}^{n}+G\beta \gamma \;mole\;fraction^{n}},$$

with n=3, $K_{d}=0.00014$, and Gβγ mole fraction calculated using the Gβγ density and lipid head group area of 6.6 × 10^−7^ μm^2^. Based on recent measurements in the lab, the $K_{d}$ reflects an approximately fourfold higher affinity of GIRK for Gβγ than reported in ref. 22.

The unroofed membrane for each montage was manually segmented and the membrane area calculated based on the pixel size of the montage and the fraction of montage occupied by the unroofed membrane. The sum of individual open probabilities ${\mathrm{Po}}_{i}$ over all GIRK channels divided by the unroofed membrane area gave the open probability density, *NPo*/*Area*.

To calculate *NPo*/*Area* for random distributions of M2R, the locations of M2R were first randomized, maintaining a constant number of proteins, using the Gold In-and-Out software (16). The distribution analysis described above was then performed for these randomly distributed M2Rs to identify any cluster centroid coordinates and number (if random clusters exist). Then, the Gβγ field and open probability density was calculated as described above.

To calculate *NPo*/*Area* when GIRK channels were randomly distributed, GIRK channels were first randomized in the footprint of the unroofed membrane using the Gold In-and-Out software (16). The random GIRK channels were then mapped onto the Gβγ field calculated from the measured M2R distribution and *NPo*/*Area* was calculated as described above.

To calculate *NPo*/*Area* when both M2R and GIRK channels were randomly distributed, GIRK channels and M2Rs were both randomized in the footprint of the unroofed membrane, a Gβγ field was calculated, and *NPo*/*Area* calculated based on the positions of the randomly distributed GIRK channels in the Gβγ field.

To predict *NPo*/*Area* when the bias is lost between M2R and GIRK HOTS, M2R cluster centroids were first randomized over the membrane, maintaining their oligomeric states and leaving GIRK HOTS in place, and then, a new Gβγ field and value for *NPo*/*Area* was calculated.

### Summary of the simulation.

Point particles representing specific protein types undergo approximate Brownian motion on a 2-dimensional surface, steps being made iteratively at intervals Δt. When two proteins come within a defined distance of each other they merge to become oligomers, which are also represented as points on the surface, and diffuse like individual proteins. In Δt an oligomer (*nmer*) can undergo a dissociation reaction, becoming an (*n−1)mer* and a single protein unit with probability proportional to a dissociation rate constant k, a geometric factor (a function of n) and Δt, k being unique to each protein type. Thus, proteins and oligomers diffuse and undergo association and dissociation. For a given initial particle number of each type, diffusion coefficient, capture radius, and Δt, interaction strengths are encoded by the k for each interaction type. For example, for a field containing M2R and GIRK there are 3 unique k values, one for the homotypic (*i-i*) M2R interaction, one for the homotypic (*i-i*) GIRK interaction, and one for the heterotypic M2R-GIRK interaction. These 3 values are adjusted to approximate the cluster size distributions and biases observed experimentally. We emphasize that this simulation is based on a set of kinetic rules rather than an equilibrium energy landscape and therefore we do not ensure that stationarity corresponds to equilibrium. The clusters generated in this simulation approximate HOTS distributions (defined using equilibrium aggregation theory in the accompanying paper (1)) and are referred to as HOTS in the figures.

### Details of the Simulation.

The code was written using MATLAB (MathWorks). Two kinds of particles representing M2R (at 2 μm^−2^) and GIRK (at 3 μm^−2^) or β1AR (at 1 μm^−2^) and GIRK (at 3 μm^−2^) were randomly distributed on a 10 μm × 10 μm field. At each time interval Δt (0.025 s) in the simulation particles in the field are displaced along x and y by a distance selected from a normal distribution with mean 0 and standard deviation $\sqrt{2D\Delta t}$, $D=0.1\;\mu m^{2}\;/\;sec$ (19). The same diffusion rule applies to oligomers, which are also represented as point particles. Association occurs if two particles (or oligomers) are separated less than 100 nm (capture radius association probability 1.0). In this manner M2R clusters, GIRK clusters, and M2R-GIRK coincident clusters are formed. Dissociation occurs according to the following rules. In each Δtan M2R protein inside an M2R *nmer* has a probability (see below) to dissociate from the *nmer* to form a monomer and an (*n−1)mer*. The dissociation probability is $k\sqrt{n}\Delta t$, where k is a dissociation rate constant for M2R, n the number of proteins in the *nmer*. Whether a protein dissociates in Δt is assessed using a pseudo random number and the dissociation probability. Once an M2R dissociates it is moved 100 nm away from its original position in a random direction. The same dissociation rule applies for a GIRK protein inside a GIRK *nmer*, but with a unique k describing GIRK dissociation. The same rule applies to the dissociation of M2R (or GIRK) from an M2R-GIRK coincident cluster. M2R and GIRK coincident clusters can also dissociate from each other in Δt with a probability given by $(k\cdot \Delta t{)}^{n1n2}$, where $\left(k\cdot \Delta t\right)$ is the probability that a complex of one M2R and one GIRK will dissociate in Δt, k the M2R-GIRK dissociation rate constant and n1 and n2 the M2R and GIRK *nmer* sizes of the coincident cluster. β1AR and GIRK simulations are carried out like M2R and GIRK simulations but with a unique k for β1AR dissociation. The positional relationship between GIRK and β1AR is unknown, but for the purposes of asking whether β1AR activates GIRK we assume it is the same as for M2R and GIRK.

Protein cluster size distributions were first calculated after 20,000 simulation steps and then every 5,000 steps. Distributions resulting from step 20,000 and step 70,000 were very similar. Graphs in Fig. 6 and *SI Appendix*, Fig. S1 correspond to k (M2R-M2R dissociation) 0.76 s^−1^, k (GIRK-GIRK dissociation) 2.0 s^−1^, k (β1AR-β1AR dissociation) 0.76 s^−1^, k (M2R-GIRK dissociation, β1AR-GIRK dissociation) 36.4 s^−1^ To analyze the individual roles of *i-i* and *i-j* interactions (Fig. 6*E*) capture radius association probabilities were individually set to 0. After each simulation, Gβγ fields and open probability densities were calculated as described above. The simulations reached stationarity within seconds (*SI Appendix*, Fig. S2). Cluster size distributions were the same whether clusters grew by addition of monomers alone or by addition of monomers and fusion of oligomers (*SI Appendix*, Fig. S3). The model makes many approximations but illustrates the basic concepts underlying dynamic connectivity.

Graphs and figures were produced using Origin(Pro), Version 2023 (OriginLab Corporation, Northampton, MA) and Adobe Illustrator (Adobe Inc. 2021).

## Supplementary Material

Appendix 01 (PDF)

Movie S1. Diffusion of M2Rs and GIRK channels in a continuous field. M2R and GIRK can each self-assemble reversibly to form higher order transient structures (HOTS) and with each other to form coincident HOTS as described in Methods. Blue, red, and yellow circles represent M2R, GIRK channel, and coincident HOTS; the circle area is proportional to the number of proteins in a HOTS or coincident HOTS. In the simulation, the total M2R density is 2 *μm^−2^* and total GIRK density 3 *μm^−2^*, near the densities measured in HL-1 cells.

## Data Availability

All study data are included in the article and/or supporting information.
