# Supplementary material for: Higher-order transient structures and the principle of dynamic connectivity in membrane signaling
Source: Proc Natl Acad Sci U S A. 2024 Dec 31;122(1):e2421280121. doi: 10.1073/pnas.2421280121 (PMC11725812; doi:10.1073/pnas.2421280121)
Supplement: Supplementary file 1 — Appendix 01 (PDF) [file pnas.2421280121.sapp.pdf]

PNAS

Supporting Information for

Higher order transient structures and the principle of dynamic connectivity in membrane signaling

Yuxi Zhang[1,2] and Roderick MacKinnon[1,2]
[1]Laboratory of Molecular Neurobiology and Biophysics, The Rockefeller University, New York, United States. 2Howard Hughes Medical Institute, The Rockefeller University, New York, United States.

Roderick MacKinnon
Email: mackinn@rockefeller.edu

This PDF file includes:

Figures S1 to S3
Legend for Movie S1

Other supporting materials for this manuscript include the following:

Movie S1

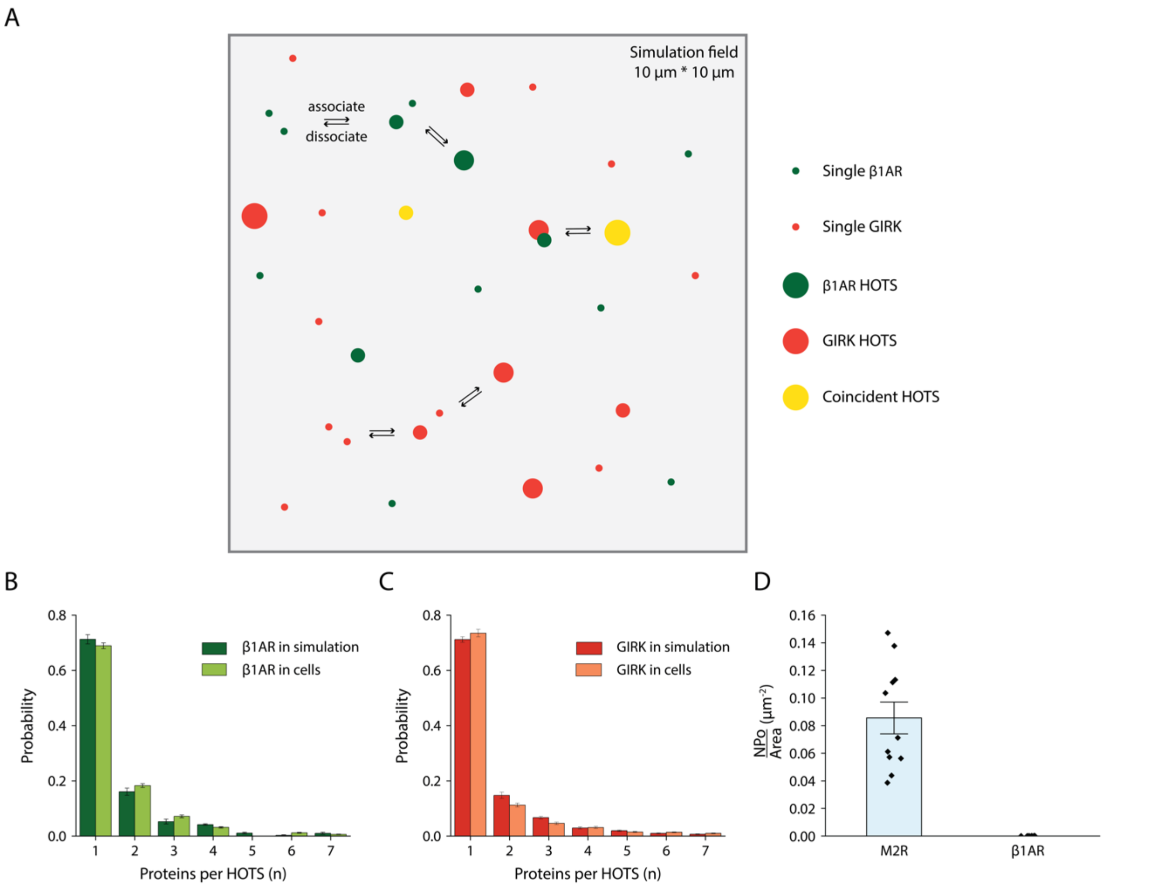

Fig. S1. Influence of β1AR on GIRK channel activation in simulation. (A) Schematic of the simulation with green, red, and yellow circles corresponding to β1AR, GIRK channel, and coincident higher order transient structures (HOTS); circle size proportional to the number of proteins in a HOTS or coincident HOTS. In the simulation, all the particles randomly diffuse with a diffusion coefficient of 0.1 $\frac{\mu m^2}{sec}$. The total β1AR density is 1 μm^{-2} and the total GIRK density is 3 μm^{-2}. (B) Normalized (meaning sum of probabilities equals 1.0) β1AR HOTS size distributions in HL-1 cells (labeled with 18 nm gold particles) or simulation. Data represent means and standard errors from 11 electron microscope montages or 11 simulations. (C) Normalized GIRK HOTS size distributions in HL-1 cells (GIRK from the M2R double-labeled montages) or simulation. Data represent means and standard errors from 17 electron microscope montages or 11 simulations. (D) Open probability density, $\frac{\mathrm{N\ Po}}{\mathrm{Area}}\,(\mu m^{-2})$, calculated from the simulation with M2R or β1AR. The Gβγ field was calculated for each simulation and open probability density was then calculated from the Gβγ field. In the simulation, the Gβγ generation rate of β1AR is one tenth that of M2R. Symbols show individual outcomes from 11 simulations (symbols) with bar height and error bars showing the mean and standard error (0.086 ± 0.012 μm^{-2} and 0.000019 ± 0.000008 μm^{-2}) from the 11 simulations.

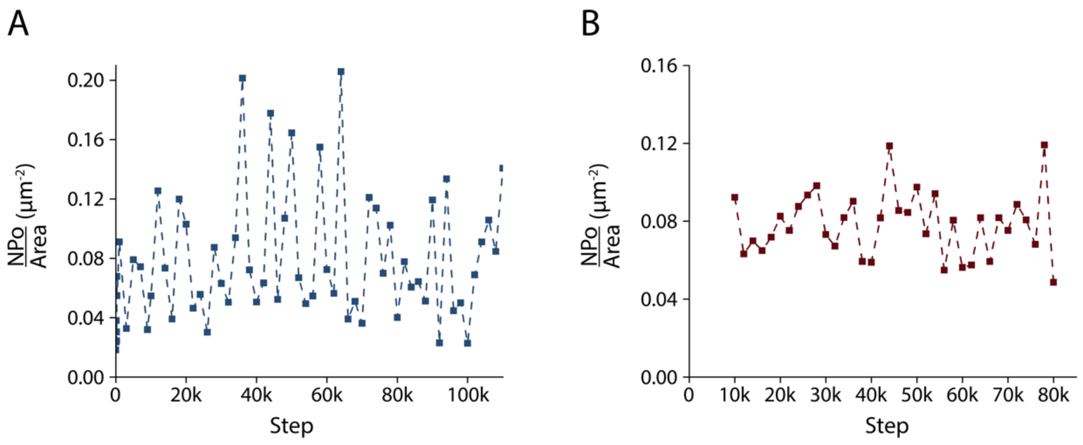

Fig. S2. Time evolution of open probability density, $\frac{N\,Po}{Area}$ (μm^{-2}) calculated using the simulation conditions shown in Fig. 6A. (A) Evolution of a single simulation trajectory. $\frac{N\,Po}{Area}$ (μm^{-2}) is calculated every 50 steps for the first 8 data points and then every 2000 steps for the remaining data points. The time interval per step $\Delta \boldsymbol{t}$ is 0.025 s. The simulations reached stationarity within seconds. The fluctuations reflect the relatively small number of proteins in a 10 μm x 10 μm area. (B) The average value of $\frac{N\,Po}{Area}$ (μm^{-2}) from 5 independent simulations from step 10,000 to step 80,000 to estimate fluctuations of the mean in a membrane with a larger area.

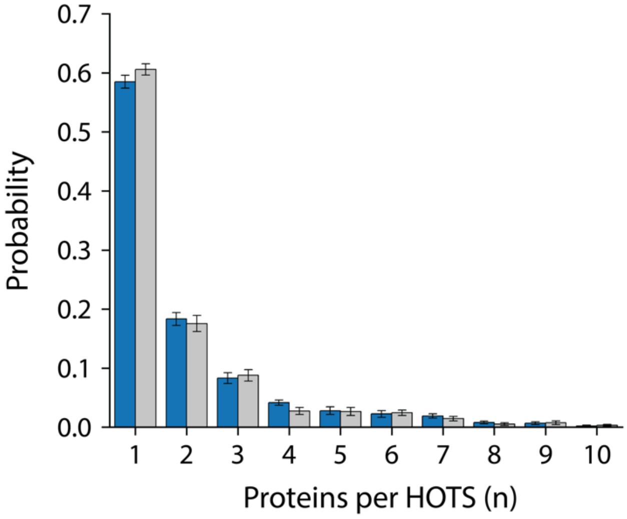

Fig. S3. Normalized M2R cluster size distributions are similar whether clusters grow by the addition of monomers alone (grey) or by the addition of monomers and fusion of oligomers (blue). Data represent means and SE from 11 independent simulations as described in Methods.

Movie S1 (separate file). Diffusion of M2Rs and GIRK channels in a continuous field. M2R and GIRK can each self-assemble reversibly to form higher order transient structures (HOTS) and with each other to form coincident HOTS as described in Methods. Blue, red, and yellow circles represent M2R, GIRK channel, and coincident HOTS; the circle area is proportional to the number of proteins in a HOTS or coincident HOTS. In the simulation, the total M2R density is 2 μm^{-2} and total GIRK density 3 μm^{-2}, near the densities measured in HL-1 cells.
